# Chemical Composition of Nanoporous Layer Formed by Electrochemical Etching of p-Type GaAs

**DOI:** 10.1186/s11671-016-1642-z

**Published:** 2016-10-04

**Authors:** Youcef A. Bioud, Abderraouf Boucherif, Ali Belarouci, Etienne Paradis, Dominique Drouin, Richard Arès

**Affiliations:** Laboratoire Nanotechnologies Nanosystèmes (LN2)—CNRS UMI-3463, Institut Interdisciplinaire d’Innovation Technologique (3IT), Université de Sherbrooke, 3000 Boulevard Université, Sherbrooke, J1K OA5 Québec Canada

**Keywords:** GaAs nanostructures, Porous GaAs, Electrochemical etching, Cathodoluminescence

## Abstract

We have performed a detailed characterization study of electrochemically etched p-type GaAs in a hydrofluoric acid-based electrolyte. The samples were investigated and characterized through cathodoluminescence (CL), X-ray diffraction (XRD), energy-dispersive X-ray spectroscopy (EDX), and X-ray photoelectron spectroscopy (XPS). It was found that after electrochemical etching, the porous layer showed a major decrease in the CL intensity and a change in chemical composition and in the crystalline phase. Contrary to previous reports on p-GaAs porosification, which stated that the formed layer is composed of porous GaAs, we report evidence that the porous layer is in fact mainly constituted of porous As_2_O_3_. Finally, a qualitative model is proposed to explain the porous As_2_O_3_ layer formation on p-GaAs substrate.

## Background

GaAs nanostructures are becoming more widespread in many applications including optoelectronic devices [1], solar cells [2], light-emitting diodes (LEDs) [3], tunnel field-effect transistors (TFETs) [4], thermoelectric devices [5], and biosensing [6]. Top-down and bottom-up methods are two approaches used to produce GaAs nanostructures by a variety of physical [7, 8], chemical [9], and electrochemical etching techniques [10]. GaAs nanostructures can be obtained in different forms and shapes including quantum wells [11], nanowires [12], quantum dots (QDs) [13], quantum dot molecules [14], quantum rings (QRs) [15], nanodiscs [16], coupled ring/disks [17], nanopillars [18], nanoholes [19], and nanopores [20]. Electrochemically formed porous GaAs nanostructures are particularly attractive for many applications due to their unique nanoscale properties and high surface-to-volume ratio. Furthermore, the applications of porous GaAs are very broad ranging from antireflective coating for GaAs solar cells [21], virtual substrate for InGaAs due to its weak elastic properties [22], and temporary carrier to reduce the weight of the solar cell by layer transfer processes [23, 24]. It was even shown that porous GaAs are good candidates to obtain fast response to humidity sensing [25]. So far, the porous morphologies obtained from the electrochemical etching of p-doped GaAs are quite different from that of the n-doped type. For n-GaAs, the material pore density, pore dimension, and layer structure depend on the doping density and the crystallographic orientation of the wafer. High aspect ratio triangular pore arrays along the (111) crystallographic direction have been observed by several groups in n-GaAs [26] and [27]. However, for p-GaAs, uniformly distributed mesopores are obtained. The argument is that holes are the majority carriers in p-type substrates and are certainly omnipresent, which will lead to a uniform dissolution even without backside illumination [28]. It is generally believed in the literature that electrochemical etching of p-GaAs leads to porous GaAs formation as it produces nanometric crystallites [29]. However, until now, no detailed analysis of the chemical composition of such layers has been reported. In this work, we report such an analysis by using various characterization techniques to reveal the chemical nature of the porous p-GaAs layer. The spatial and spectral distribution of cathodoluminescence (CL) in bulk and porous samples is presented. Energy-dispersive X-ray spectroscopy (EDS) and X-ray photoelectron spectroscopy (XPS) analysis were performed to confirm the chemical composition. Finally, X-ray diffraction (XRD) was used to determine crystallographic properties of the porous layer.

## Methods

Porous GaAs layers are formed by electrochemical etching on highly doped p-type wafers (Zn-doped, resistivity = 2.10^−3^ ohm.cm) with (001) crystal orientation supplied by AXT. After immersing the GaAs wafer in an electrolyte consisting of a 49 % HF/water solution, the etching process is activated via direct current. The characteristics of the surfaces are extracted from SEM images using a Zeiss scanning electron microscope, operated from 1 to 20 kV and equipped with a field emission gun. Cross-section images are obtained by cleaving the samples. The bulk porosity is determined through gravimetric measurements, where the sample is weighed by a high precision scale (±0.1 mg) before (*m*_1_) and after (*m*_2_) the etching process. Afterwards, the wafers are introduced for 10 s in H_2_O:H_2_O_2_:H_3_PO_4_ (140:2.5:1) in order to selectively remove the porous layer. The remaining mass of the substrate is obtained by weighing the sample again (*m*_3_). The porosity (*P*) is given by *P*% = (*m*_1_ − *m*_2_)/(*m*_1_ − *m*_3_). The cathodoluminescence (CL) spectra and images are acquired at room temperature, in the same SEM setup as the one used for imaging the layers. Our CL system, in association with a spectrometer, allows monochromatic CL (GATAN MonoCL2) imaging as well as acquisition of CL spectra on localized spots of a sample with a spectral resolution of 0.5 nm. The accelerating voltage used in the CL characterization is 20 KeV. Surface chemistry is analyzed by XPS Kratos Axis Ultra DLD with monochromatic Al Kα (1486.7 eV) X-ray source and an oval beam size of 300 × 700 μm in diameter. The electron take-off angle is fixed at 60° and the vacuum pressure is below 10^−9^ Torr during spectra data acquisition. Survey XPS data are acquired over 1200 eV with pass energy of 160 eV and a resolution of 1 eV. High-resolution XPS spectra are obtained at Ga 3d, As 3d, O 1 s, F 1 s, and C 1 s with a pass energy of 20 eV. Binding energies, peak areas, and atom concentration ratios are obtained using CasaXPS. The chemical composition of the exposed surface is verified also by energy-dispersive X-ray (EDX) spectroscopy. Powder X-ray diffractograms have been measured with a Philips X’Pert diffractometer. Samples are prepared simply by crushing porous layer and measured on a standard single crystal.

## Results and Discussion

### Electrochemical Calibration and Morphology

**Fig. 1:**
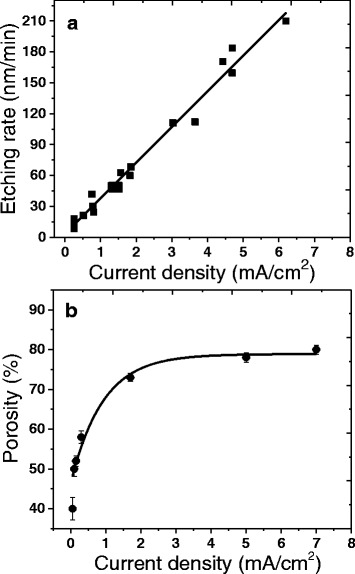
Etching rate (**a**) and porosity evolution (**b**) versus the current density of anodically etched p-GaAs in a 49 % HF solution

**Fig. 2:**
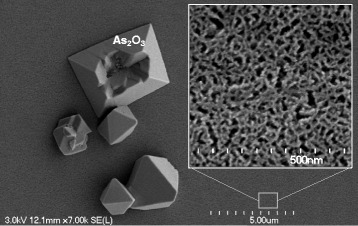
Planar-view SEM image of anodically etched p-GaAs layer formed in 49 % HF solution at 2 mA/cm^2^. The *inset* shows high-resolution SEM image on the porous surface

Calibrations of the porous layer parameters such as porosity and etching rate have been done by varying the current density in the electrochemical process, all other conditions being the same. Figure 1a, b illustrates the dependence of the porosity and the etching rate on the current density. Similarly to porous Si and Ge [30, 31], the etching rate increases linearly with increasing current density. The porosity initially increases rapidly, until it gradually saturates at around 80 %. During the drying process, cracks appear over the entire surface of the sample. The origin of the cracks can be attributed to the high mechanical stresses generated when the water is evaporated [32, 33]. The SEM image of GaAs after porosification is shown in Fig. 2; we can see the presence of micro-crystallites, with sharp edges, dispersed on the surface of the porous layer and having sizes of several hundred nanometers that are attributed to As_2_O_3_ precipitates as it was already observed by Smeenk et al. [34]. The resulting clusters show the pyramidal and prismatic morphology with a preferential orientation along the (111) plane corresponding to tiny octahedra As_2_O_3_ crystals [35, 36]. High-resolution SEM image of the porous surface shows a randomly and uniformly distributed mesopores structure. The nanocrystallite size is estimated to be in the range of 10–20 nm.

### Luminescence of Porous Layer

**Fig. 3:**
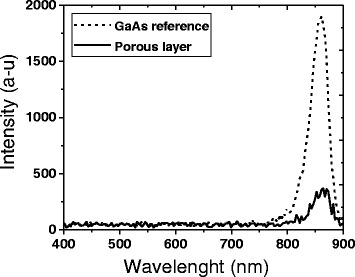
Cathodoluminescence spectra at room temperature for the p-GaAs reference substrate and the porous p-GaAs with 20 keV at room temperature formed in 49 % HF solution at 2 mA/cm^2^

**Fig. 4:**
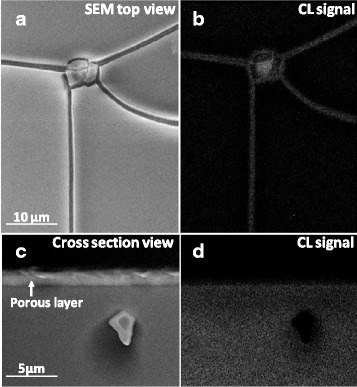
SEM and CL micrographs taken from the top surface (**a**, **b**) and cross section (**c**, **d**) of porous p-GaAs with 20 keV at room temperature formed in 49 % HF solution at 2 mA/cm^2^

Because of the small crystallite size (in the range of 10–20 nm) that composes the porous layer, it is expected that such layer should emit radiation above the GaAs bandgap due to the quantum confinement effect [37]. In order to investigate this effect, we performed cathodoluminescence microanalysis of a GaAs sample before and after the porosification process. CL spectra from the reference sample show luminescence in the near infrared region with a maximum intensity at 860 nm as shown in Fig. 3. The peak is correlated to the interband recombination process of exited charge carriers across the direct bandgap of p-GaAs (1.43 eV) at room temperature [38]. After the porosification process, the CL intensity was considerably attenuated and no detectable shift in the peak was observed despite the low crystallite size of the porous material. This indicates that no quantum confinement effect is created within the crystallites. Which is in contrast with the results obtained by Lockwood et al. who observed luminescence in the infrared (~840 nm) and green (~540 nm) PL peak wavelengths after anodic treatment in HCl solution that are consistent with an assignment to quantum confinement effects in GaAs micro- and nanocrystallites, respectively. The previous observations suggest that the size distribution of the pores correlated qualitatively with the intensities and the positions of the PL peaks, whereas variations in the chemical composition at the surface show no systematic correlation [39, 40]. The drastic decrease in the GaAs peak intensity could be due to different reasons, such as (i) the quenching of the CL due to the surface state, (ii) fractional amorphization of material during anodization, and (iii) change in chemical composition. The later assumption will be verified in the following paragraphs. However, less attention has been paid to the investigation of the direct correlation between luminescence properties and morphology features of the porous GaAs layer. The morphology and CL characteristics of porous layers reported on the spatial mapping of the intensity (Fig. 4) reveal luminescence only around the location of cracks formed during the porosification process. The cross-sectional CL maps also show no luminescence coming from the porous layer. These results suggest that the CL signal is coming from the underlying bulk GaAs material, which is exposed directly in the cracked areas or by the penetration of some electrons through porous layer at high accelerating voltage (20 KeV). The porous layer does not seem to generate detectable luminescence.

### Chemical Compositions

**Fig. 5:**
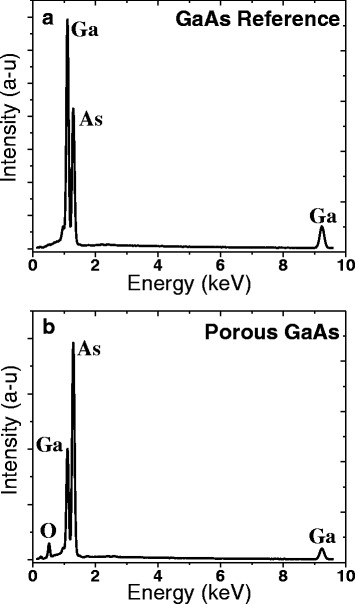
EDX spectra of p-GaAs sample (**a**) and porous p-GaAs layer formed in 49 % HF solution at 2 mA/cm^2^ (**b**)

**Fig. 6:**
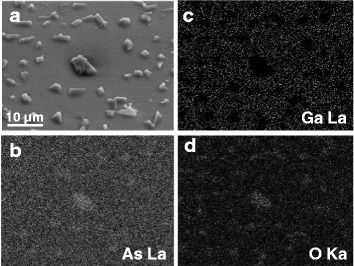
EDX mapping of a porous p-GaAs layer formed in 49 % HF solution at 2 mA/cm^2^. **a** The top view SEM image and the EDX element maps for **b** As, **c** Ga, and **d** O elements

The absence of size-related luminescence could be due to non-radiative recombinations or to a drastic change in the chemical nature of porous material during anodization. We have investigated this question by verifying that the porous material stoichiometry remained similar to that of GaAs after porosification. Figure 5 shows EDX spectra of the GaAs bulk reference sample and the porous GaAs sample. We notice that the ratio between the Ga- and As-related emissions is changed for the porous layer. It seems that the porous material is enriched with As relative to the reference sample and there is an oxygen-related signal that appears as well. A similar EDX study shows a high decrease of Ga peak on crystalline particles during the anodic dissolution of GaAs in HF solution [41, 42]. An oxygen peak is also present in the porous spectrum indicating a high concentration of oxygen in the film. The EDX mapping of the top of the porous sample confirms the presence of clusters with only As and O contents as shown in Fig. 6. Since the EDX spectrum integrates signals coming from the whole field of view, it is unclear if the oxygen is present in the porous material, or only in the clusters on the surface.

**Fig. 7:**
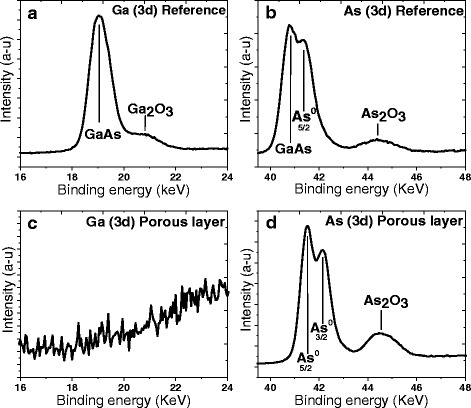
Ga 3d and As 3d XPS spectra of p-GaAs reference (**a**, **b**) and porous p-GaAs samples (**c**, **d**) immediately after anodization in 49 % HF solution at 2 mA/cm^2^ showing that only the As and O are present in detectable quantities

In order to determine the quantitative composition of the porous GaAs layer, we have performed XPS measurements before and after anodization. High-resolution spectra from the As 3d and Ga 3d regions are shown in Fig. 7. A small amount of Ga_2_O_3_ and As_2_O_3_ is detected on the surface of the reference sample (Fig. 7a, b) due to air exposure [34]. The Ga and As signals appear in the reference sample and are coming from both the base GaAs material as well as the oxides (Ga_2_O_3_ and As_2_O_3_). The XPS analysis of the porous material (Fig. 7c, d) clearly shows that it consists only of As and arsenic oxide elements. The Ga signal has completely disappeared. However, the Ga presence in EDX spectra is due to the sensitivity of this technique that offers to probe the sample within few microns contrary to XPS measurement with depth analysis of about 8–10 nm. The elemental concentration of the porous layer taken at different surface locations is summarized in Table 1 and indicates that only As and O atoms are present in detectable quantities. This is due to the preferential dissolution of Ga during anodization, leaving a significantly higher As/Ga ratio in the porous layer. This is in agreement with the work of Steer et al. who have shown that Ga dissolved faster than arsenic during polarization in phosphoric acid [41].

**Table 1:** Chemical composition of the porous sample by XPS measurements taken at the surface

Name	Binding energy (eV)	FWHM (eV)	% at conc	% mass conc
O 1 s	531,2	2,605	23,46	6,15
Ga 3d	23,2	2,335	0,58	0,66
As 3d	42,2	2,758	75,96	93,19

### Electrochemical Mechanisms

**Fig. 8:**
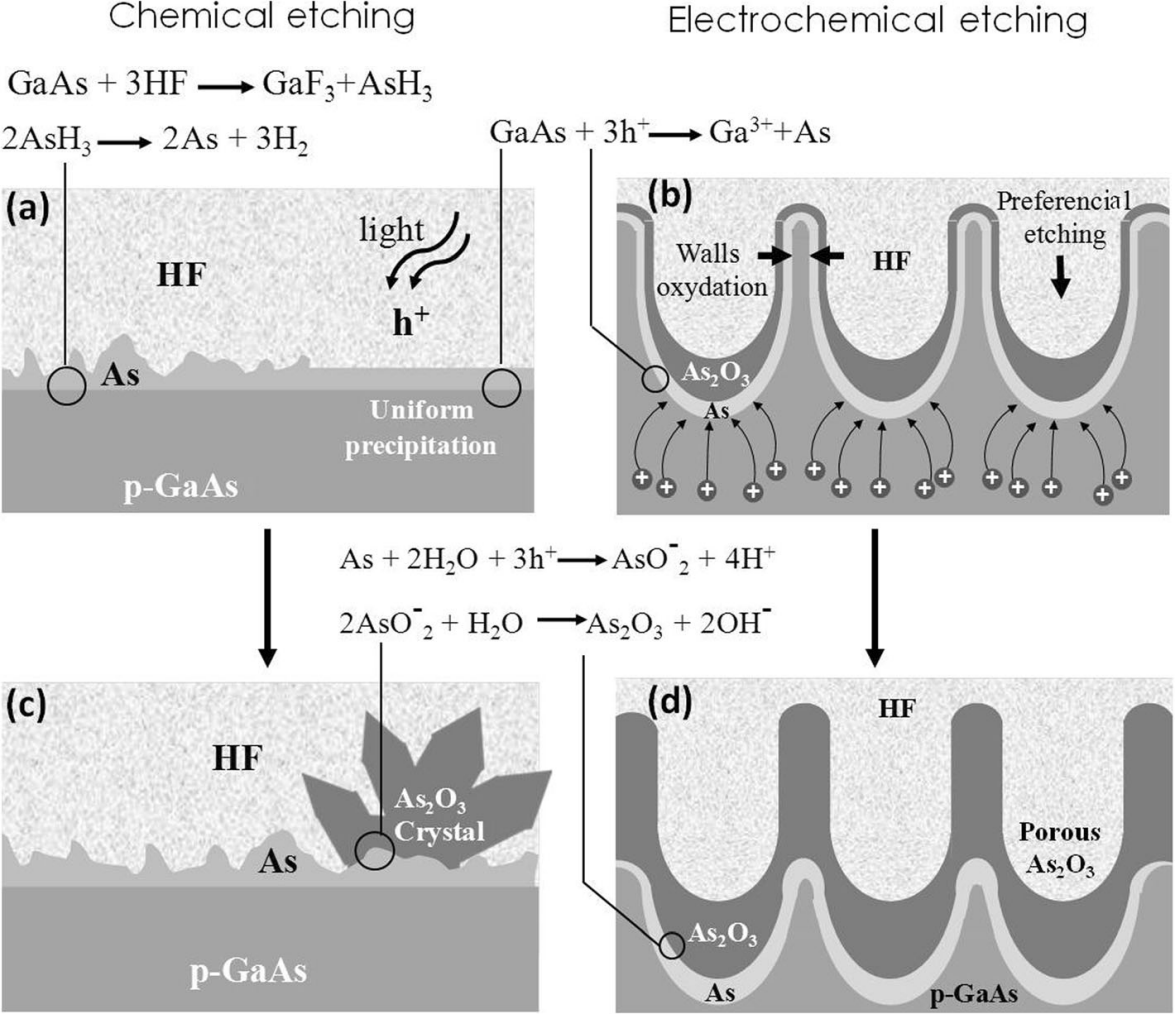
Chemical (**a**, **c**) and electrochemical (**b**, **d**) etching mechanism of GaAs in HF solution

In order to explain the behavior of GaAs transformation to porous arsenic oxide under anodic bias, we propose a qualitative model based on the decomposition of compound semiconductors in contact with electrolytes [43]. Figure 8 schematically illustrates the etching mechanism of GaAs in a HF solution. The electrochemical dissolution of semiconductors depends essentially on the symmetry of bi-functional etching agents [44, 45]. The GaAs dissolution in HF is similar to InP in HCl [46]. The first step involves a synchronous exchange of bonds: Ga-F and As-H bonds replace the GaAs surface bonds [47]. The surface becomes H-passivated if the crystal was As-terminated or F-passivated if the crystal was Ga-terminated [48]. This reaction increases the bond polarity of goshawks atoms. Since the surface atoms are doubly bonded to the bulk in the (001) orientations and triply bonded to the bulk in the (111) orientations [49], two and three GaAs bulk bonds must be broken to remove each atom from the lattice following:

1 $$ \mathrm{Ga}\mathrm{As}+3\mathrm{H}\mathrm{F}\to \mathrm{Ga}{\mathrm{F}}_3+\mathrm{A}\mathrm{s}{\mathrm{H}}_3 $$

Note that the last GaAs bond should be broken without substitution to produce GaF_3_ and AsH_3_.

The second step consists in the formation of arsenic. The elemental arsenic (As) can be obtained by two ways: (i) chemically: from the arsine molecules AsH_3_ as shown in Fig. 8a. In this case, a non-uniform As layer was obtained due to the difficulty of the nucleation [34] following

2 $$ 2\mathrm{A}\mathrm{s}{\mathrm{H}}_3\to 2\mathrm{A}\mathrm{s}+3{\mathrm{H}}_2 $$

(ii) Electrochemically: by involving holes (Fig. 8b) or protons (Fig. 8a) in a redox reaction following a three-hole mechanism proposed for the etching of GaP [50] and InP [46]. For p-GaAs, an accumulation of majority charge carriers (holes) takes place at the surface [51]. In aqueous HF solutions, three holes are required to dissolve one GaAs entity in which only the Ga element is oxidized. This highlights the increasing of the etch rate with the current density applied as shown in Fig. 1. In this case, a uniform As layer was obtained due to the uniform injection of holes following

3 $$ \mathrm{G}\mathrm{aAs}+3{\mathrm{h}}^{+}\to \mathrm{G}{\mathrm{a}}^{3+}+\mathrm{A}\mathrm{s} $$

It was observed that a brown film of As was formed on the electrode during electrochemical etching [34]. The same behavior was revealed in the case of InP etching in HCl solution with a high content of phosphorus but not indium [46]. We suggest that the injection of the holes anodically speeds up this selective etching which explains the low Ga concentration obtained previously in the porous layer.

The last step consists in the formation of As_2_O_3_. The presence of water in the electrolyte solution oxidizes elemental As following

4 $$ \begin{array}{l}\mathrm{A}\mathrm{s}+2{\mathrm{H}}_2\mathrm{O}+3{\mathrm{h}}^{+}\to \mathrm{A}\mathrm{s}{{\mathrm{O}}^{-}}_2+4{\mathrm{H}}^{+}\\ {}2\;{{\mathrm{As}\mathrm{O}}^{-}}_2+{\mathrm{H}}_2\mathrm{O}\to {\mathrm{As}}_2{\mathrm{O}}_3+2{\mathrm{O}\mathrm{H}}^{-}\end{array} $$

Figure 8c shows 3D structures of As_2_O_3_ obtained on the electrode surface following reaction (2) or reaction (3) via As transformation to As_2_O_3_ by convective diffusion [52]. Additionally, the chemical formation of As_2_O_3_ and the porosification process can take place simultaneously, which produces a porous arsenic oxide layer as shown in Fig. 8d. The low crystallite dimension is obtained by the nucleation and the coalescence of the oxide layer formed on the wall pores which will stop the reaction.

### Structural Analyses

**Fig. 9:**
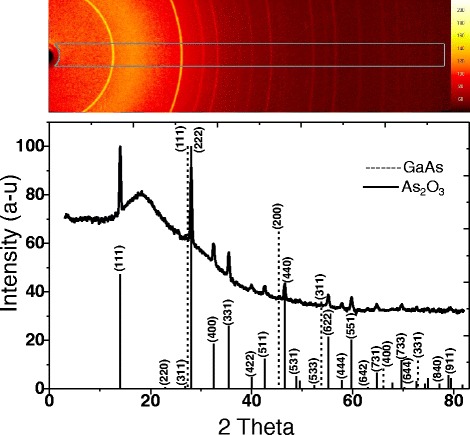
Powder XRD of porous p-GaAs layer formed in 49 % HF solution at 2 mA/cm^2^ which corresponds to As_2_O_3_ crystal

The last measurement was performed by powder XRD to investigate the crystal structure of the porous film. Figure 9 shows the diffractogram of a crushed porous layer obtained by scraping and grinding the porous sample to ensure that the measurement takes place on the porous layer and not on underlying GaAs substrate. The single-crystal X-ray diffraction measurement is obtained by mounting the crushed porous particles on the goniometer. All other diffraction peaks can be identified as the standard cubic of arsenolite structure (As_2_O_3_) with a lattice constant of 11.06 A°. No peak from other expected phases such as GaAs (zincblende) or As_4_O_6_ (cubic) are observed within the detection limit of our technique. Effectively, all the peaks correspond to the reflections from (111), (222), (400), and (331) planes of the cubic phase of As_2_O_3_ [53]. Hence, the XRD patterns show that the As_2_O_3_ film is of a single phase. The intensity of the (111) peak is very strong and its width at half maximum is relatively narrow, indicating a good crystallization state through a large crystallites size.

## Conclusions

The nanostructural and chemical nature of porous layers obtained by the anodic etching of p-type GaAs has been investigated. No detectable CL has been attributed to the chemical change of porous GaAs during anodization. Chemical analysis shows that the porous material is depleted of Ga atoms and contains significant amounts of oxygen. Structural data from XRD show that the porous material is composed essentially of crystalline As_2_O_3_. An electrochemical process is proposed to explain such a behavior.

## Abbreviations

CL: Cathodoluminescence
EDX: Energy-dispersive X-ray spectroscopy
XPS: X-ray photoelectron spectroscopy
XRD: X-ray diffraction
